# The MCTOT app: A publicly available tool for statistical cycle-to-threshold analysis and inference of informative but uncertainly determined qPCR data

**DOI:** 10.1371/journal.pone.0330729

**Published:** 2025-09-02

**Authors:** Wei Zhuang, Jessica Liu

**Affiliations:** 1 Division of Bioinformatics and Biostatistics, National Center for Toxicological Research, U.S. Food and Drug Administration, Jefferson, Arkansas, United States of America; 2 Department of Statistics, Rice University, Houston, Texas, United States of America; OMICS, PERU

## Abstract

As a common experimental technique, qPCR (Quantitative Real-time Polymerase Chain Reaction) is widely used to measure levels of nucleic acids, e.g., microRNAs and messenger RNA. While providing accurate and complete data, researchers have inevitably encountered uncertainly determined qPCR data because of intrinsically low amounts of biological material. The presence of incomplete or uncertain qPCR data challenges interpretation accuracy. This study presents a web application that integrates two sophisticated statistical methods – a flexible regression approach and a two-group hypothesis testing technique – to enhance the accuracy and robustness of qPCR data analysis with informative but uncertainly determined observations. To demonstrate the versatility and efficacy of our MCTOT (Multi-Functional Cycle-To-Threshold Statistical Analysis Tool) application, this study presents two distinct examples employing two-group hypothesis testing. The first example delves into an analysis of pathogens in wastewater, an area gaining increasing relevance for public health surveillance. The second example illustrates an application in the realm of liquid biopsy, a rapidly evolving field in disease diagnostics, monitoring, and early treatment. Moreover, the application’s process is further exhibited through another liquid biopsy example, wherein the flexible regression method is employed to detect the hemolysis effect on a molecular target. These examples demonstrate the tool’s capacity to not only identify significant differences between groups but also to quantify the effect size, a crucial aspect in biomedical research. The MCTOT web application stands as a pioneering step toward empowering researchers to harness the full potential of qPCR data, especially when dealing with informative but uncertainly determined observations. It also paves the way for further development of web-based tools that adhere to the refined CTOT (Cycle-To-Threshold) methodology, opening new avenues in qPCR data analysis and interpretation. The developed application can be accessed online through Shinyapps.io at https://ctot.shinyapps.io/bioinformatics/ for open access.

## 1 Introduction

Quantitative Real-time Polymerase Chain Reaction (qPCR) is a molecular biology tool with applications in gene expression analysis, pathogen detection, liquid biopsies, among many others. Its importance has been recently highlighted during the COVID-19 pandemic for the detection of SARS-CoV-2 RNA in wastewater and clinical samples. qPCR is utilized to determine the concentration of a molecular target through continuously monitoring and measuring of a fluorescent signal that directly corresponds to the amount of the target molecule in the PCR exponential phase. The reach of the fluorescence threshold in qPCR reactions may be treated as an event of interest as reaching the fluorescence threshold not only indicates the presence of the target but also enables its quantification. The qPCR cycle quantification (Ct) value is commonly defined as the cycle at which the reaction curve reaches the fluorescence threshold level. A lower Ct value shows a high amount of gene expression while a higher Ct value shows a low amount of gene expression. In addition, qPCR experiments may have maximum assay specific Ct cutoff values, such as 32 or 40 [1,2]. Thus, besides complete data, researchers have also observed informative yet incomplete data, e.g., in liquid biopsies and community-level wastewater samples [2–5] due to the nature of qPCR technology and biological materials.

Informative association and differential changes may involve absent, low-level, poorly expressed, or completely unexpressed nucleic acids in liquid biopsies and public health surveillance. Such low abundance of nucleic acids results in incomplete or uncertain data in qPCR experiments. This issue can pose significant challenges to the foundational assumptions of prevalent statistical techniques used for qPCR data analysis like t-tests, which require or assume complete datasets for analysis. When faced with incomplete qPCR data, researchers or investigators from different disciplines may diverge in their analytical tactics or strategies, commonly selecting from three distinct approaches: the Complete-Observation method (CO), the Maximum Cycle (MC) method, or Choosing Not to Analyze (CNA) the targets with incomplete data. The CO method excludes incomplete observations, leading to a reduced sample size [1,2,6]. In the MC method, incomplete observations are substituted with a predetermined maximum Ct value, such as 32 or 40. This approach, while straightforward, can potentially distort the distribution of Ct data [2,6]. On the other hand, the CNA approach entirely omits targets with incomplete observations from the analysis. Each of these methods has its drawbacks, with CO potentially reducing statistical power due to smaller sample sizes, and MC risking data bias through oversimplified or inappropriate imputation [1,2,6]. A lack of a scientifically and statistically sound method and tool to analyze and interpret patterns with incomplete or uncertain qPCR data may hinder biomarker detection, pathogen detection, and gene expression analysis.

Zhuang et al. [2] introduced a robust and sensitive statistical method of CTOT (Cycle-To-Threshold) to help leverage qPCR technology and increase the power to detect differential changes that may involve low number of nucleic acids, e.g., microRNAs in liquid biopsy. The underlying principles and theories of the CTOT method were also introduced for qPCR data analysis in general [2,7,8]. Through this introduction, the groundwork was effectively laid for what would be recognized as the CTOT methodology. The CTOT methodology that focuses on robustness and accommodates incomplete or uncertain qPCR data can be used in association testing likewise. This holistic and multi-functional CTOT methodology for qPCR data analysis can enhance analytical and interpretation capabilities of qPCR technology across diverse scientific applications.

A range of desktop and web-based qPCR software tools, such as Qiagen RT^2^ Profiler PCR Data Analysis, have been designed and used to analyze complete datasets from PCR arrays [1,9]. Incomplete data must be imputed or removed before inferential statistical analysis using these software tools, as the tools are designed to handle complete datasets [1,9]. The removal and imputation may bring unnecessary bias and decrease statistical power [2,6]. We implemented CTOT in an online software tool. Complementing existing software to conduct a scientifically and statistically sound analysis with qPCR data beyond a maximum Ct cutoff value, our online MCTOT (Multi-Functional Cycle-To-Threshold Statistical Analysis Tool) software streamlines the implementation of the CTOT methodology for various applications.

## 2 Materials and methods

### 2.1 A R package for web applications

The creation of interactive web applications within the R statistical computing environment was made possible by the introduction of the R Shiny package [10]. Other R packages may be seamlessly integrated with the R Shiny package. While R applications created by the R Shiny package can be executed locally, these applications are often deployed and operated in a cloud-based environment, such as Shinyapps.io. In this study, the R Shiny package was integrated with other R packages, including coin, dplyr, broom, shinyjs, DT, survival, and summarytools R packages. The resulting application is made available online at https://ctot.shinyapps.io/bioinformatics/ via Shinyapps.io for open access.

Our R Shiny application is an advanced tool designed to refine or enhance the analysis of qPCR data. It offers users a versatile platform to upload their data files, select specific variables of interest, and set a Ct cutoff. A key feature of our application is its user-friendly interface that simplifies and streamlines complex analytical processes using the CTOT methodology. By allowing users to set their own Ct cutoffs, the application provides a tailored approach to qPCR data analysis, accommodating different research or scientific contexts. This feature is particularly important in studies where the determination of a precise Ct cutoff can significantly impact the interpretation and relevance of qPCR results. In this study, we used three examples to illustrate the usage of the application.

### 2.2 Three examples based on real-life studies

Three illustrative examples were sourced from real-life studies. These examples showcase how the MCTOT application may be applied in various research scenarios using qPCR techniques to determine concentration changes in nucleic acids, often faced with inevitably incomplete/uncertain qPCR data due to intrinsically low amounts of biological materials.

The first example represents the scenarios where qPCR was used to investigate the presence of pathogens, such as SARS-CoV-2, in wastewater [5]. The second and third examples illustrate the scenarios where qPCR was utilized in liquid biopsies to analyze body fluids like serum and urine for biomarker detection, facilitating early diagnosis and guiding treatment strategies [2,4]. The experiments in all three examples were conducted with reliable pre-PCR procedures and real-time qPCR processes [2,4,5].

The first two examples were used to illustrate two-sided CTOT robust nonparametric two-group hypothesis testing. The first example referred to a study using qPCR to detect and evaluate SARS-CoV-2 genomes in wastewater inflow from two urban regions in Arkansas [5]. Wastewater surveillance of SARS-CoV-2 is an important tool to follow the dynamics of COVID-19 in communities. It provides information regarding viral shedding from both asymptomatic and symptomatic individuals, and it could serve as an early warning for potential surges in COVID-19 cases. In the published study [5], viral RNA was extracted from influent wastewater using a MagMAX™ Microbiome Ultra Nucleic Acid Isolation Kit (Applied Biosystems, Foster City, CA). The levels of SARS-CoV-2 RNA were measured by qPCR with a set of TaqMan™ RT‑PCR assays (TaqMan™ 2019-nCoV Assay Kit v1, Applied Biosystems) targeting three different viral genes that encode open reading frames for 1ab polyprotein (ORF1ab), surface glycoprotein (S-protein), and nucleocapsid phosphoprotein (N-protein). Additionally, these assays also include a specific test for an endogenous control (Human RNase P RPPH1 gene). The qPCR analysis was performed on a QuantStudio™ 7 Flex (Applied Biosystems). For illustration of the MCTOT application, the first example focused on a quarterly analysis of concentration differences between S-gene and N-gene. Pepper mild mottle virus (PMMoV) was selected to normalize the Ct values, removing unwanted variance in the Ct values, such as inevitable differences in starting biological materials for qPCR processes [5]. PMMoV is frequently used as an internal reference in SARS-CoV-2 wastewater studies [5,11,12] as it is an abundant and often stably expressed plant RNA virus found in human feces [13,14]. Specifically, the concentrations of SARS-CoV-2 S-gene and N-gene were compared in the third quarter (July through September) of 2021 from the Adams Field, a wastewater treatment facility in Little Rock, AR.

The second example pertained to a study on the effect of a 28-day co-exposure to nephrotoxic doses of melamine (MEL) and cyanuric acid (CYA) on serum microRNAs levels in rats [4]. It was also used to illustrate two-sided CTOT robust nonparametric two-group hypothesis testing. In the published study [4], researchers reported the impacts of dietary intake of MEL and CYA on circulating microRNAs in rats. Literature indicated that exposure to MEL and CYA could lead to kidney damage, including in humans [4,15]. Circulating microRNAs showed potential as valuable biomarkers for early detection and effective medical intervention. In the published research [4], blood was processed to serum, and the amount of free hemoglobin in the serum was measured using spectrophotometry to assess hemolysis. The entire RNA content, including microRNAs, was extracted from the serum using a miRCURY RNA Isolation Kit for Biofluids (Exiqon, Vedbaek, Denmark). The target microRNAs were measured using qPCR with an ABI 7900HT Fast Real-Time PCR System and TaqMan miRNA assays (Applied Biosystems by Life Technologies, NY, USA). To ensure quality control throughout the RNA extraction, reverse transcription, and qPCR processes, five microRNA spike-ins (Exiqon) were incorporated at various stages. The normalizer, which had been experimentally validated prior to the data analysis, was miR-342-3p [4]. In the second example, the levels of serum microRNA miR-210-3p in male rats were compared between the control group and the exposure group that had been dosed with 240 ppm MEL and CYA in feed [4].

The third example sourced from FDA/NCTR internal data on the impact of hemolysis on circulating cell-free microRNAs. It was utilized to illustrate CTOT flexible semiparametric regression for liquid biopsies, contributing to the depth and scope of the illustration of the MCTOT application. Hemolysis of blood samples, which is breakdown of red blood cells, could be a potential issue or constraint during sample collection or preparation in liquid biopsies as the release of the contents of red blood cells may contaminate and substantially impact the plasma or serum samples with erythrocytes-derived nucleic acids, such as microRNAs [2,4]. It is important to determine whether potential normalizers and targets of interest may be affected by hemolysis in qPCR-related liquid biopsies, as hemolysis can contaminate samples with intracellular contents, artificially alter molecular levels, introduce analytical variability, compromise the normalization process, and ultimately impact decision-making and research reproducibility. In the third example, the level of hemolysis was estimated by measuring the absorbance at 414 nm (A414 nm) of the serum samples using a Nanodrop 1000 spectrophotometer. Crucially, the effect of hemolysis on the microRNA mmu-let-7d-5p in serum from female NCTR F344 rats was assessed after normalization using the geometric mean of three stable microRNA references (miR-30e, miR-146a, and miR-139-5p) [2,4,16,17]. The data on the microRNAs have not yet published but included here to illustrate the application. The details of the normalization selection process are beyond the scope of this illustration. Interested readers may refer to literature for further information [16,17].

Rigorous quality control prevented technical errors in the three examples [2,4–6]. Using the standard definition of an outlier – any value outside the expected range – no data points in the exemplar datasets were flagged, while reference ranges for these emerging study areas are still being established [18]. Consequently, these datasets are suitable for demonstrating the MCTOT app, which supports statistical inference of data generated from error-free pre-PCR preparation and reliable real-time qPCR reactions performed on low-copy-number biological materials, such as extracellular nucleic acids. Interested readers may see the Data Availability Statement for data access.

### 2.3 The CTOT methodology implemented in the MCTOT application

The CTOT methodology is unique in that it incorporates essential characteristics of qPCR data and time-to-event statistical methodology [2]. It aims to incorporate high-quality qPCR data from all subjects, regardless of whether the data points are below, meet, or exceed the Ct cutoff value, such as 32 or 40. The CTOT methodology embraces normalization, a crucial step in qPCR data analysis, and employs a binary variable to indicate whether the Ct value is smaller than the Ct cutoff value that is determined prior to the data analysis, such as, 32 or 40. The CTOT methodology also incorporates the principles of time-to-event data analysis within its framework, treating the reach of a fluorescence threshold in qPCR reactions as a significant event. This approach allows for analyzing qPCR data as cycle-to-threshold data, reflecting interest in both whether the fluorescence threshold is reached by the assay-specific maximum qPCR cycle and the exact amplification cycle where the fluorescence threshold is achieved within the predefined qPCR cycle limits. It is worth noting that censoring due to the presence of a Ct cutoff value is more comparable to administrator censoring in survival or time-to-event studies than other types of censoring, such as participant dropout. Although normalization that adjusts Ct values based on an internal reference does not change the fundamental nature of the censoring mechanism in qPCR data, it can influence the specific points at which normalized data may be censored. Thus, the CTOT methodology incorporates relevant principles of time-to-event data analysis and suits the typical characteristics and requirements of qPCR data analysis [2].

This study focuses on CTOT flexible semiparametric regression and CTOT robust nonparametric two-group hypothesis testing, both of which incorporates characteristics of qPCR data and relevant principles of time-to-event data analysis. As mentioned in our previous publication [2], the CTOT robust nonparametric two-group hypothesis testing method employed the exact Fleming-Harrington method and provided improved power for detecting differential changes between groups compared to the traditional methods of MC, CO, and CNA. The two-group CTOT method is a nonparametric method and does not rely on many of the common parametric assumptions, such as normality and the proportional CTOT rate assumption, where the CTOT rate is defined as the conditional rate to reach the fluorescence threshold [2]. The proportional CTOT rate assumption refers to the assumption that the effect of the explanatory variable on the CTOT rate is constant or proportional over qPCR cycles. Like other nonparametric methods, the two-group CTOT method focuses on the significance of the results (i.e., whether an effect exists) rather than the magnitude of the effect. If users are keen on understanding effect sizes and the proportional CTOT rate assumption can be reasonably assumed, they may opt for the CTOT flexible semiparametric regression that adapts the Cox proportional hazards model to analyze qPCR data.

### 2.4 Data security and user privacy

The developed application can be accessed online through Shinyapps.io. Shinyapps.io does not provide persistent storage for user-generated data between application sessions. As a result, any changes to the file system are lost when the application becomes idle and enters a sleeping state, ensuring that no user data is retained between sessions.

### 2.5 Statistical justification and sensitivity tests

While the MCTOT app was primarily illustrated using three real-life datasets – the SARS-CoV-2 genome in wastewater, liquid biopsy, and hemolysis impact – these were selected for their strong relevance to real-world, multifunctional applications. We also expanded our validation efforts to include both simulated datasets and a complementary publicly available external dataset, further showcasing MCTOT’s broader applicability and generalizability. Larger simulated datasets with 1000 replications and an additional human colorectal cancer (CRC) dataset were used for power and sensitivity analysis, providing statistical justification, and ensuring power and robustness of the analyses [2,6,19]. The CRC dataset from a published study provided valuable insights into a wider range of biological and environmental contexts [6,19]. In general, researchers can use the app to analyze any dataset for comparison or association analysis with inherently low amounts of biological material, whether simulated or real-world.

### 2.6 Comparison with an existing qiagen data analysis tool

We performed side-by-side comparisons with a traditional qPCR tool, i.e., Qiagen RT^2^ Profiler PCR Data Analysis, to demonstrate that MCTOT produces comparable or improved results when handling incomplete data [9]. This comparison included an assessment of hosting/availability, application scope, and extensibility.

### 2.7 Terminology clarification

We standardized the use of “Ct” throughout this paper, except when referring to the app interface. Both the “Cq” and “Ct” appear in the app interface to ensure that the analytical tool remains accessible and user-friendly for researchers, reflecting the variety of the terms they use.

### 2.8 Ethics statement

This study re-analyzed de-identified qPCR datasets and performed power analyses using simulated data only. No new human participants or live animals were involved; therefore, Institutional Review Board (IRB) and Institutional Animal Care and Use Committee (IACUC) approval were not required.

## 3 Results

**Fig 1:**
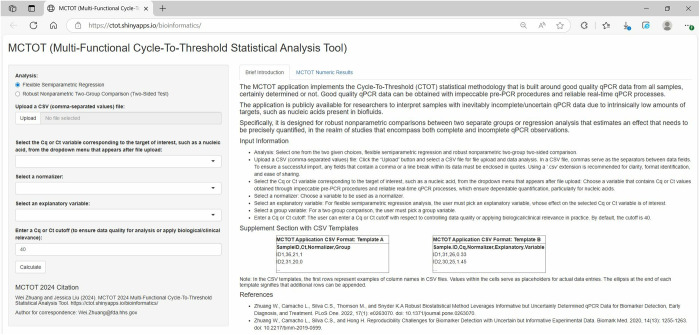
The MCTOT app interface. It is the main interface. Users may press browser’s Refresh/Reload (Ctrl + R/Command + R) to return all inputs to their defaults.

We implemented the statistical CTOT methodology in a R Shiny app named MCTOT, enabling analysis of high-quality qPCR data from all samples, whether certainly determined or not. The application has undergone compatibility checks with Firefox, Microsoft Edge, and Google Chrome. Fig 1 illustrates the general design of the app within a typical web browser. At the top of the interface, as depicted in Fig 1, the name of the application is given and described. In the interactive interface, on its left section, users can opt for either semiparametric flexible regression analysis or a robust nonparametric two-group comparison (Fig 1). This choice is followed by file uploading for data analysis, choosing relevant variables, and setting Ct cutoff value. The app requires uploading a file in the CSV (Comma-Separated Values) file. The file format is renowned for its widespread use and flexibility in handling tabular data [20–22]. This step is vital for the subsequent process of selecting variables and conducting data analysis within the app. The CSV file format is a plain text format where each line represents a single row of data, pertaining to a subject or record. Commas separate individual data values within each row. The first row should contain concise, descriptive column names. To ensure a successful import, any fields that contain a comma or a line break within its data must be enclosed in quotes. Each subsequent row represents a single sample, with data for each sample organized horizontally. Specifically, the Ct values of the target of interest as well as the normalizer, necessary for both analysis types, must be all numeric. Moreover, semiparametric regression necessitates a numeric explanatory variable, while the robust nonparametric two-group comparison requires a binary group variable, which may be labeled as 0 and 1 or as “group1” and “group2”. The default value of the Ct cutoff is 40. The user has the option to modify this value to suit their specific study requirements by either using the upper arrow to increase the value or the lower arrow to decrease it. Alternatively, the user may enter a precise number directly into the textbox. During analysis, any values meeting or surpassing the user defined Ct cutoff will be considered as uncertain or incomplete. If a sample’s Ct value is classified as undetermined, the respective Ct value in the input CSV dataset should be a number that is equal to or exceeds the user defined Ct cutoff. Additionally, the input dataset may include other variables for user convenience or preferences, although those additional variables will not be directly utilized in the analysis. For example, it is generally advised to include a variable dedicated to sample names or IDs for traceability and good practice.

**Fig 2:**
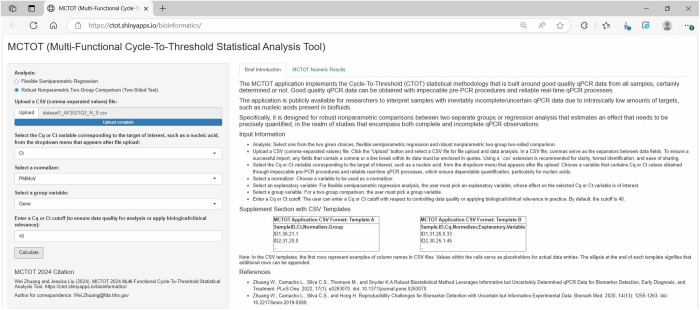
Input file, variables, and values of example 1 with SARS-CoV-2 wastewater data. Specifying “Ct” as the “Cq/Ct” variable; choosing “PMMoV” as the normalizer; selecting “Gene” as the group variable; and setting the “Cq/Ct” cutoff at 40. Here, “Cq/Ct” denotes the interchangeable use of Cq and Ct, reflecting the nomenclature differences in guidelines and applications.

**Fig 3:**
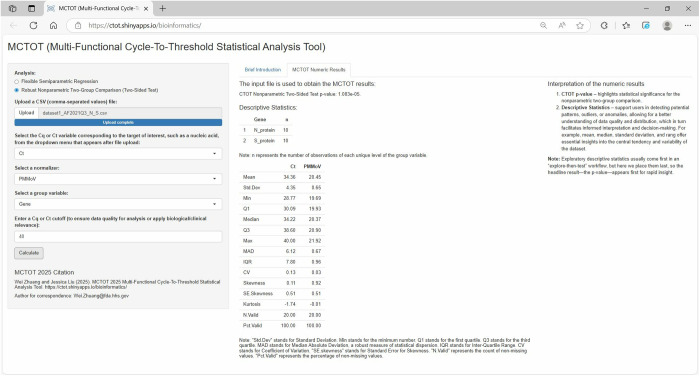
Results of example 1. It is regarding analysis of SARS-CoV-2 wastewater data using the MCTOT application.

Through the app, the user can obtain CTOT statistical results as well as descriptive statistics [2]. Fig 2 illustrates how a file can be uploaded into the app, and how variables can be selected for a robust nonparametric two-group comparison. In the quarterly analysis of the SARS-CoV-2 genome in wastewater influent using the web application for a robust nonparametric two-group comparison, the user can start by selecting the analysis type, inputting the data, which includes Ct values for S-gene and N-gene, gene group variable information that differentiates between observations of the S-gene and those of the N-gene, as well as a normalizer, which is PMMoV. Cq/Ct denotes the interchangeable use of Cq and Ct, reflecting differing nomenclatures in guidelines and applications. Upon running the comparison with a click on the “Calculate” button, the application processes the data, considering the gene group and normalizing factor, to robustly compare the measured genomic segment concentrations specific to the S-gene and N-gene. The outcome of this analysis is presented in the form of a p-value, which indicates the statistical significance of the differences observed between the gene groups. A low p-value (typically p-value < 0.05) would suggest a significant difference in measured concentrations and provide critical insights into viral genetics, conditional upon the premise that the samples are of reasonable and consistent quality. As shown in Fig 3, the CTOT p-value and descriptive statistics are reported in the online MCTOT Numeric Results section, while guidance on interpreting those descriptive and inferential statistics can be found in the online Interpretation of Numeric Results subsection. In the first example, the CTOT p-value was 1.083e-05, which suggested a significant difference in the measured concentrations of S-gene and N-gene. It suggested that a comparative analysis of the S-gene and N-gene within the viral genome has revealed discrepancies in their concentration in the samples. These genes play pivotal roles in SARS-CoV-2’s replication cycle, with the S-gene being integral for host cell entry and the N-gene crucial for RNA packaging and replication processes. The observed differences in the two gene parts could stem from an important biological reason, provided that the samples have reliable and reasonable quality. An earlier study [5] demonstrated the identification of two mutations (L452R and P681R) using amplicon RNA sequencing and qPCR assays utilizing probes aimed at specific mutations in the S-gene during the third quarter of 2021 from Adams Field as described in the Materials and Methods section. Details regarding the RNA sequencing and allele-specific qPCR assays are beyond the purview of this paper. Readers seeking further information are encouraged to consult the earlier publication [5]. In addition, the descriptive statistics like mean, median, variance, and range of the variables in the input dataset provide a comprehensive view of the overall nature and patterns of the data. Thus, the MCTOT application, exemplified in its application to wastewater SARS-CoV-2 data, emerges as a powerful tool, adept at facilitating mutation detection and enhancing epidemiological surveillance and public health decision-making.

**Fig 4:**
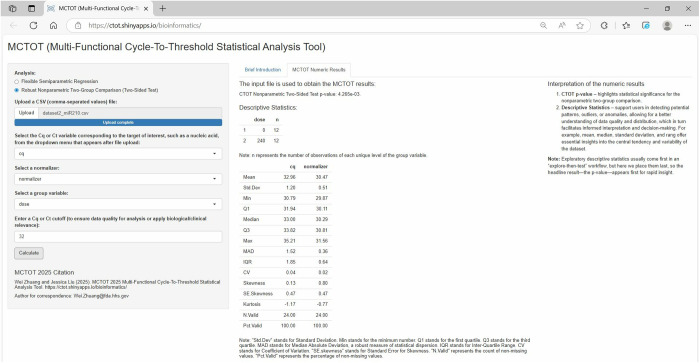
Input file, variables, values, and results of example 2 with liquid biopsy data. Specifying “cq” as the “Cq/Ct” variable; choosing “normalizer” as the normalizer; selecting “dose” as the group variable; and setting the “Cq/Ct” cutoff at 32. Here, “Cq/Ct” denotes the interchangeable use of Cq and Ct, reflecting the nomenclature differences in guidelines and applications.

In the second example involving liquid biopsies, users can effectively use the web application to perform a robust nonparametric two-group comparison to evaluate an exposure group against a control group. The exposure group was 240 ppm MEL- and CYA-dose group. The published study used miR-342-3p as a normalizer variable due to its consistent expression across various samples [4]. Should the Ct cutoff value be 32, the user can update the Ct cutoff from the default value, which is 40, to the new value of 32 in the application. Upon entering all details into the web application as shown in Fig 4, the app undertook a nonparametric statistical analysis and provided the CTOT p-value of 4.265e-03, which suggested a significant difference in the concentrations of miR-210-3p between the control and exposure groups. Additionally, the web application generated descriptive statistics of the input data, including parameters like mean, median, standard deviation, and range. These statistics provide a deeper insight into the input data.

**Fig 5:**
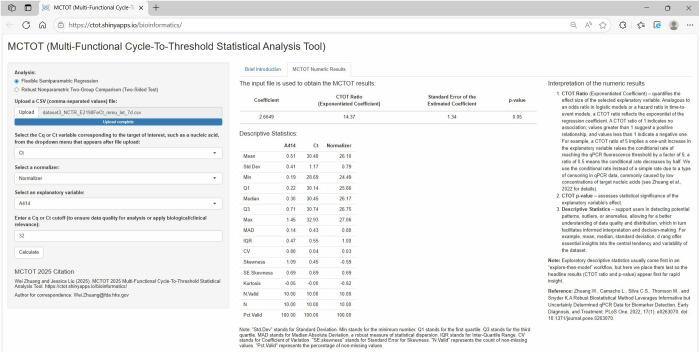
Input file, variables, values, and results of example 3 with liquid biopsy data. Specifying “Ct” as the “Cq/Ct” variable; choosing “Normalizer” as the normalizer; selecting “A414” as the explanatory variable; and setting the “Cq/Ct” cutoff at 32. Here, “Cq/Ct” denotes the interchangeable use of Cq and Ct, reflecting the nomenclature differences in guidelines and applications.

In the context of liquid biopsy analysis, researchers can also employ the web application to conduct semiparametric regression, providing valuable insights into the relationship between a molecular marker and a relevant explanatory variable. In the third example presented (Fig 5), the user enters critical information, including Ct values, a normalizing factor, and an explanatory variable, the web application utilizes the CTOT semiparametric regression technique to model the non-linear association between the Ct values and hemolysis while the normalizing factor is miR-342-3p as mentioned in the Materials and Methods section. As previously noted, Cq/Ct denotes that the terms Cq and Ct are interchangeable, highlighting the variation in nomenclature observed across different guidelines and practical settings. Hemolysis in the dataset was quantitatively measured by determining the absorbance of serum samples at 414 nm, a specific wavelength indicative of hemoglobin presence, using a Nanodrop 1000 spectrophotometer, yielding a mean absorbance of 0.51, with a standard deviation of 0.41, and observed values ranging from a minimum of 0.19 to a maximum of 1.45. The output includes crucial statistical metrics such as p-values and effect sizes for the explanatory variable, offering a comprehensive understanding of hemolysis impact on the molecular marker under investigation, mmu-let-7d-5p. The estimated CTOT ratio for the hemolysis effect on mmu-let-7d-5p was 14.37, as shown in Fig 5. The associated p-value was 0.05, aligning was the typical significance level of 0.05. Thus, there was an indication that hemolysis might affect the level of mmu-let-7d-5p; in particular, a one unit increase in A414 nm reading might increase the rate to reach the fluorescence threshold by a factor of 14.37. In other words, an increase in A414 nm reading was potentially associated with a lower Ct value. Research with larger sample sizes or studies in different but relevant contexts may help clarify the biological significance of this association. Additionally, the web application provides descriptive statistics of the input file, furnishing researchers with a summary of the central tendencies and variability in the data. General guidance on interpreting those descriptive and MCTOT inferential statistics can be found in the online Interpretation of Numeric Results subsection, as shown in Fig 5.

We performed associated power analyses for our three real-world illustrative examples in alignment with the widely used commercial data analysis software NCSS, which reports associated power for certain tests [22]. Consistent with the NCSS approach, we assumed that the necessary population parameters for power analysis, such as means and standard deviations, were equivalent to the corresponding sample estimates. Two-sided alternative hypotheses and a significance level of 0.05 were used in power analysis, mirroring the settings of the three illustrative analyses with MCTOT. For each of the first and second examples (Fig 3 and Fig 4), a total of 1000 simulation replicates were performed in R [2] (additional settings in S2 Text). The associated power values were 100% and 46% for the analysis performed in MCTOT, while the values were 100% and 13% for the MC method, which set the censored values equal to the user-specified Ct cutoffs of 40 and 32 for the first and second examples, respectively [2]. Each replicate preserved the original sample sizes of 10 and 12 per group, respectively. The associated power for the third example was estimated at 90%. It is important to note that the examples were intended to illustrate the application of MCTOT. The diversity in the associated power values across the examples reflects the inherent variability in real-world scenarios, particularly in the contexts of sensitivity analyses and retrospective analyses.

MCTOT was developed following current best practices, including unit testing, integration testing, functional testing, and load testing. The first example, a novel application, and the second example, which was previously analyzed in the published paper introducing the CTOT methodology, both utilized the robust nonparametric two-group comparison (two-sided test) analysis type [2]. In the second example, the results generated by the MCTOT app matched those reported in the original publication [2]. The third example applied the flexible semiparametric regression analysis type. In this case, the MCTOT app performed normalization and applied the user-specified Ct cutoff; when normalization and the Ct cutoff were manually performed and the processed data were subsequently analyzed using NCSS with the flexible semiparametric Cox regression, the results matched those produced by MCTOT within standard rounding conventions.

Table 1 compares our MCTOT app with ReadqPCR, NormqPCR, and Qiagen RT^2^ Profiler PCR Data Analysis in terms of hosting/availability, application scope, and extensibility [9,17]. Both the MCTOT app and the Qiagen tool can handle two-group comparisons [9]. In general, the Qiagen tool uses a parametric t-test, MCTOT uses a nonparametric method for two-group comparisons and semiparametric for regression [9]. For benchmarking, we applied our MCTOT app and the Qiagen tool to the publicly available miR-96 discovery dataset, which had 10 controls and 20 CRC early-stage patients [19]. The Qiagen tool substituted Ct 50 values with the tool’s maximum Ct cutoff 40 and offered only an equal-variance t-test (Table 1), whereas Sun et al. [19] retained the 50 values in their analysis. Building on significant findings in the discovery cohort, where miR-96 exhibited a p-value of 0.031 when retaining Ct 50 values, Sun et al. [19] proceeded to validation and found plasma levels of miR-96 remained significantly elevated in CRC (p-value = 0.003) in an independent set of 187 CRC patients and 47 healthy controls. However, that initial p-value 0.031 was inherently fragile – modest shifts in Ct handling (capping at 40) readily eliminated the signal. With the Ct cutoff at 40 and the same normalizer used by Sun et al. [19], both Qiagen’s equal-variance t-tests and MCTOT yielded non-significant p-values (0.33 and 0.38, respectively), whereas the original authors report a p-value equal to 0.031 when using a Ct cutoff of 50. The loss of signal may be expected, since in the discovery cohort only one CRC sample had an original Ct < 40 whereas all other control and early-stage CRC measurements were recorded as 50. Moreover, Sun et al. [19] supplemented their t-tests with Mann-Whitney tests, a non-parametric method, illustrating that nonparametric approaches were necessary for qPCR data inference.

**Table 1:** Benchmark of our MCTOT app with ReadqPCR, NormqPCR, and Qiagen RT^2^ profiler PCR data analysis.

Benchmark	MCTOT Shiny App	ReadqPCR	NormqPCR	Qiagen RT^2^ Profiler PCR Data Analysis
Hosting and availability	Freely accessible Shiny web application requiring no local installation, with openly available source code permitting local, optional, offline deployment	Offline use in R	Offline use in R	Proprietary web application requiring user registration
Application scope	Non-parametric two-group comparisons and simple semiparametric regression for intrinsically low-input qPCR data, e.g., assays of nucleic acids in biofluids	Data ingestion and structuring, bridging raw qPCR data from diverse formats into a standardized Bioconductor R object, facilitating downstream Bioconductor analyses, (e.g., normalization via NormqPCR)	Optimal selection of stable reference genes and normalization of qPCR data in a Bioconductor R object	Using two-sample, unpaired, two-tailed t-tests assuming equal variance to assess significance, this streamlined qPCR pipeline covers data import, quality control, normalization, fold-change calculations, and plotting –restricted to Qiagen-provided methods, which do not include those offered by MCTOT or NormqPCR
Extensibility	Using the open-access MCTOT source codes, users can add more group comparisons or regression analyses beyond those bundled with MCTOT	Its open-source nature allows extension or modification to suit novel research needs or accommodate specific experimental requirements	As an open-source package, its codebase can be customized to integrate additional workflows or normalization methods as needed	Its statistical functionality is limited to Qiagen’s offerings – for example, group comparisons use only parametric, two-sample, unpaired, two-tailed t-tests assuming equal variance, with no option for other statistical methods, such as regression or nonparametric methods

## 4 Discussions

The main purpose of the MCTOT application is to enhance the statistical analysis and interpretation of informative yet uncertainly determined qPCR data through an intuitive, user-friendly R Shiny web interface. The potential benefits of MCTOT lie in its ability to extract interesting scientifically insights—even from samples with inherently low target concentrations—thereby maximizing data utility and enhancing productivity in molecular research, clinical, diagnostic, and regulatory applications. This study has demonstrated the MCTOT application’s capabilities using practical examples from liquid biopsies and the SARS-CoV-2 genome in wastewater influent. These examples were chosen due to their relevance in current research and their ability to showcase the application’s versatility in handling different types of biological data. Liquid biopsies represent a challenging matrix due to the typically low concentration of target nucleic acids, while wastewater influent offers a complex environmental sample, demonstrating the application’s ability to handle diverse sample types. Reliable pre-PCR procedures and real-time qPCR processes are integral to the effective deployment of our application, in line with the idea that the best possible data quality results in valuable statistical results and facilitate research reproducibility.

A challenge encountered in conventional qPCR data analysis is the presence of uncertain or incomplete qPCR data, which is often the result of naturally low target concentrations, as is typical in wastewater or biological fluids like blood. This uncertainty can pose significant hurdles in data interpretation, making robust analytical tools like our application essential. Our application is adept at handling such data, providing researchers with the means to derive meaningful insights even from samples with low target concentrations. It showcases the potential of the CTOT methodology for robust and comprehensive analysis of qPCR data. It opens avenues for users to further develop R Shiny applications using methods aligned with the CTOT methodology, such as the method that incorporates covariates in addition to the explanatory variable of interest. However, while the R language and the R Shiny package can technically accept a variety of input formats, MCTOT presently standardizes on CSV input files. CSV files, being plain text with clear comma delimiters and no hidden metadata, are easy for software to read and provide transparent and consistently structured data representations. Most modern qPCR software tools that can analyze amplification data—both commercial (e.g., QuantStudio Design and Analysis, Bio-Rad CFX Manager) and open-source (e.g., shinyCurves)—can export key results, such as Ct values, in CSV format [23–25]. Common tools such as Microsoft Excel can readily convert TXT, XLS, or XLSX files to CSV [26]. If direct ingestion of these formats is required, researchers may adapt the MCTOT (S1 Text) to accept them. In addition, the current build raises Shiny’s default 5 MB upload cap to 10 MB—ample for typical qPCR CSVs (< 1 MB). Local deployments can raise this limit further by adjusting Shiny’s upload size setting. They can also keep all data on-premises to meet strict compliance requirements for local data retention, while the online version provides convenient cloud access.

It is critical to emphasize that, before applying CTOT or MCTOT, we strongly recommend evaluating potential outliers or experimental errors using established methodologies described in the literature before applying MCTOT [18]. If experimental errors are suspected, researchers should consider excluding the erroneous data or repeating the experiments to ensure accurate results. Potential outliers can be flagged or re-examined through repeated measurements. Researchers may also conduct sensitivity analyses by running their analyses with and without these potential outliers. Nevertheless, we concentrate on scenarios involving best practice of qPCR technology—specifically, error-free pre-PCR preparation and dependable real-time qPCR reactions. In addition, a covariate, such as the degree of hemolysis in liquid biopsy samples, can be incorporated into a regression model. Researchers may perform a sensitivity analysis comparing regression models with and without this covariate to evaluate its statistical and scientific significance in contributing to data noise (i.e., unexplained data variance) in liquid biopsy samples that may be contaminated by hemolysis [27].

MCTOT enables the creation of an efficient, reproducible workflow for analyzing qPCR data derived from low-abundance biological materials, particularly in emerging areas. For example, shinyCurves, ΔXpress, and MCTOT are all source-available R Shiny applications [25,28]. The R shiny shinyCurves app processes amplification data from qPCR machines, while ΔXpress supports normalizer selection and visual inspection of data distributions through interactive plots [25,28]. Because MCTOT accepts any CSV containing numeric Ct values and a chosen normalizer, results exported from tools such as shinyCurves and ΔXpress can be imported directly and analyzed with MCTOT’s robust two-group test or flexible semiparametric regression, whichever suits the study design [25,28]. Researchers can also build bespoke pipelines in Python—or any computational environment that can run R—by calling MCTOT’s core statistical functions (S3 Text). For instance, users can blend MCTOT’s nonparametric test or semiparametric regression with the classical t-tests and ANOVA provided by the Python-based auto-qPCR web app, creating flexible workflows that unite both classical and assumption-light qPCR analyses while accommodating diverse data types and study contexts. These open, standardized workflows balance the financial and technical barriers of commercial software with the real-world demands of rapid, cross-lab collaboration. It contributes to broader efforts to develop accessible, interoperable tools that help researchers detect and respond to emerging public-health signals more efficiently [29,30].

In the realm of qPCR-based analytics, the importance of meticulous data analysis cannot be overstated. The use of advanced software and methodology plays a pivotal role, enabling the distinction of true signals from background noise. While alternative analysis approaches, such as Bayesian modelling and machine learning-based methods, have been explored in qPCR data analysis, they often rely on prior knowledge or extensive historical/training datasets, which may be limited in emerging research contexts [31–33]. In contrast, the present study centers on the development of a user-friendly web application that integrates a flexible regression method and two-group hypothesis testing, particularly tailored for handling uncertain data due to intrinsically low levels of biological materials (e.g., nucleic acids in biofluids) in such contexts. To contextualize existing tools, we compared the MCTOT app with Qiagen’s RT^2^ Profiler PCR Data Analysis tool and two related R packages, as shown in Table 1. Additionally, both Bio-Rad’s CFX Manager and Thermo Fisher’s QuantStudio tools emphasize instrument-specific workflows, supporting qPCR data acquisition, Ct value generation, and basic analyses such as ΔΔCt [23,24]. This comparison underscores MCTOT’s analytical strengths, particularly its ability to handle incomplete or uncertain qPCR data without imputation or data exclusion. In essence, rigorous data analysis in qPCR not only bolsters the accuracy of the findings but also enhances the confidence in the interpretations from biological and molecular investigations. Thorough analysis is indispensable for ensuring the reliability of qPCR-based diagnostics and research conclusions.

## Supporting information

S1 Text R code for the MCTOT shiny application. (TXT)

S2 Text Power analyses of the three illustrative real-world examples using the CTOT methodology. (DOCX)

S3 Text MCTOT core functions and testing scripts in R. (TXT)

## Abbreviations

A414: Absorbance at 414 nm (spectrophotometric index of hemolysis)
CNA: Choosing Not to Analyze method (omits targets with incomplete data)
CO: Complete-Observation method (excludes incomplete observations)
Ct: Cycle threshold (qPCR cycle at which fluorescence crosses the threshold)
CTOT: Cycle-To-Threshold statistical methodology
Cq: Quantification cycle (alternative term for Ct)
CYA: Cyanuric Acid
FDA: U.S. Food and Drug Administration
MC: Maximum Cycle method (imputes incomplete observations with the Ct cut-off)
MCTOT: Multi-Functional Cycle-To-Threshold Statistical Analysis Tool (a web app)
MEL: Melamine
miRNA: microRNA
N-gene: Nucleocapsid gene of SARS-CoV-2
N-protein: nucleocapsid phosphoprotein of SARS-CoV-2
NCTR: National Center for Toxicological Research, a research center of FDA
ORF1ab: SARS-CoV-2 gene that encodes open reading frames for 1ab polyprotein
PCR: Polymerase Chain Reaction
PMMoV: Pepper mild mottle virus (internal reference for wastewater studies)
qPCR: Quantitative Real-time Polymerase Chain Reaction
R: R statistical computing environment
S-gene: Spike gene of SARS-CoV-2
S-protein: the viral surface spike (S) glycoprotein of SARS-CoV-2
SARS-CoV-2: Severe Acute Respiratory Syndrome Coronavirus
