# Supplementary material for: The MCTOT app: A publicly available tool for statistical cycle-to-threshold analysis and inference of informative but uncertainly determined qPCR data
Source: PLoS One. 2025 Sep 2;20(9):e0330729. doi: 10.1371/journal.pone.0330729 (PMC12404495; doi:10.1371/journal.pone.0330729)
Supplement: S2 Text — (DOCX) [file pone.0330729.s002.docx]

*Supporting information*

S2 Text. Power Analyses of the Three Illustrative Real-World Examples Using the CTOT Methodology

Power analyses were performed for the three illustrative scenarios using the CTOT methodology [2]. Following NCSS software conventions [22], key population parameters were approximated by their sample estimates due to limited data in emerging areas. A two-sided significance level of 0.05 was applied throughout.

In the first two scenarios (Figs 3 and 4), 1,000 simulated replicates were generated for each scenario, preserving original group sizes in each replicate (10 and 12, respectively). Simulations reflected Ct uncertainty at thresholds of 40 and 32, respectively, using Simulation Type A as defined in Zhuang et al. [2]. In the scenarios, normalizer values were assumed to follow N (20.454, 0.649) and N (30.475, 0.510), respectively, and normalized Ct values were modeled with log-normal distributions. Gene and dose group effects were incorporated via coefficients of –8.46 and 2.3807, respectively, indicating group-related Ct differences, i.e., a substantial Ct decrease in the N-gene group compared to the S-gene group and an interesting Ct increase in the dose 240 group relative to dose 0.

The median simulated Ct was 34.65 (Q1 = 30.38, Q3 = 38.83) and 33.73 (Q1 = 32.52, Q3 = 34.93), which were consistent with the sample estimates for the first and second scenarios. Data are available via Figshare at <https://doi.org/10.6084/m9.figshare.28917242.v1> or <https://figshare.com/articles/dataset/Two_Simulated_Datasets_for_the_MCTOT_Paper/28917242/1?file=54140147>.

Using the MCTOT approach, the empirical power reached 100% in the first scenario and 46% in the second. In contrast, the MC method—which substitutes censored values with predefined Ct thresholds—yielded power estimates of 100% and 13%, respectively.

In the third scenario, a Cox regression on the CTOT rate achieved 90% power (α = 0.05) with 10 observations and a coefficient of 2.66 [22]. The assumed cycle-to-threshold rate of 0.9, analogous to a hazard rate, highlights CTOT’s capacity to model uncertainty in qPCR data [2]. The differing power levels illustrate the variability present in real-world data, particularly in retrospective analyses and sensitivity assessments.
